# Adult presence does not ameliorate juvenile feeding challenges in a leaf-footed bug

**DOI:** 10.1098/rsos.221291

**Published:** 2023-08-02

**Authors:** Sam Zlotnik, Christine W. Miller

**Affiliations:** ^1^ School of Natural Resources and Environment, University of Florida, 2035 McCarty Hall D, Gainesville, FL 32611, USA; ^2^ Entomology and Nematology Department, University of Florida, 1881 Natural Area Dr, Gainesville, FL 32611, USA

**Keywords:** Coreidae, generalist diet, feeding facilitation, herbivory, *Leptoglossus zonatus*, structural defence

## Abstract

Herbivores often grapple with structural defences in their host plants, which may pose especially difficult challenges for juveniles due to their underdeveloped feeding morphology. The degree to which juvenile herbivore survival is limited by structural defences as well as the strategies used to overcome them are not well understood. We hypothesized that juveniles benefit from feeding near adults because adults pierce through physical barriers while feeding, enabling juveniles to access nutrients that they otherwise could not. We tested this feeding facilitation hypothesis in the leaf-footed bug *Leptoglossus zonatus* (Hemiptera: Coreidae). Bugs were raised with an adult or a juvenile conspecific and fed a diet of pecans with or without shells. As predicted, we found that juveniles suffered greater mortality when fed nuts with shells than when fed nuts without shells. Contrary to our expectations, the presence of an adult feeding on the same nut did not lessen this effect. Therefore, the presence of an adult does not ameliorate the feeding difficulties faced by juvenile *L. zonatus,* despite evidence for feeding facilitation in related insect species. This study adds to our understanding of how host plant defences can limit the survival of even highly generalist herbivores.

## 1. Introduction

Herbivores must often overcome a range of structural and chemical plant defences to feed successfully [1,2]. Structural defences in host plants, such as trichomes, shells and latex, may be especially important in imposing limits on herbivory [3]. Yet most research on adaptations of herbivores focuses on overcoming chemical defences rather than structural ones [4,5]. Structural plant defences may pose particular challenges for juvenile herbivores due to their relatively small size and underdeveloped feeding morphology [6]. Juveniles might also have fewer feeding options than adults due to being less adept at foraging over large distances and at higher danger of predation if they do. Plant defences may therefore impose high costs on herbivores during the juvenile stage in particular (e.g. [7]). It is unclear what strategies juvenile herbivores use to feed on structurally defended host plants or the extent to which they are impacted by these defences.

Many animals use behavioural adaptations, in addition to morphological ones, to help them feed [8]. Juvenile herbivores may rely on behavioural strategies to compensate for their inadequate feeding morphology. For example, juveniles may choose to feed near larger conspecifics, such as adults, that can more easily overcome structural plant defences. When juveniles feed at the same sites as adults, they might access nutrients that they could not have accessed alone due to, for example, the feeding damage that adults can inflict upon plants [9]. This facilitation of juvenile feeding by adults is most often considered within the context of parental care [10,11]. However, parental care is not required for juveniles to capitalize on the feeding behaviours of adults.

We investigated whether feeding facilitation by adult conspecifics can increase juvenile survival in the leaf-footed bug *Leptoglossus zonatus* (Hemiptera: Coreidae). These bugs use their piercing-sucking mouthparts, or rostrums, to consume the buds, fruits and seeds of over 20 plant families [12]. Their host plants include some with hard shells or thick rinds, such as almonds [13], citrus [14] and pomegranates [12], as well as others without such barriers, such as tomatoes [15]. As *L. zonatus* is a hemimetabolous insect, nymphs (juvenile bugs) are smaller and have shorter rostrums than adults, which limits their maximum potential penetration depth [16]. Because of this limitation, nymphs may have more difficulty feeding on structurally defended host plants compared with adults.

Juvenile *L. zonatus* cannot fly as their wings do not fully develop until adulthood. Therefore, they may be unlikely to find suitable alternative host plants if they attempt to leave their natal host plant. Dispersal is also risky for nymphs as leaf-footed bugs face a wide range of predators, including ants, assassin bugs and birds [17]. In environments with low plant diversity, such as many agricultural fields, the potential benefits of dispersal are likely to be especially low compared with the potential costs. Nymphs may need to survive on whatever host plant they hatched on, regardless of feeding challenges. Indeed, other species of leaf-footed bugs often complete their entire juvenile development on the same host plant that their eggs were laid on (e.g. [18]). However, coreids also often aggregate on the same individual plants [17], which may present an opportunity for nymphs to gain easier access to nutrients by taking advantage of feeding facilitation by adults.

There is some evidence for non-parental feeding facilitation in leaf-footed bugs as well as related hemipteran insects. For example, Cenzer [9] found that juvenile *Jadera haematoloma* (Hemiptera: Rhopalidae) were more likely to survive to adulthood when feeding on seeds that had been previously fed on by adults. This facilitative effect of adult feeding was greater when bugs were raised on host plants with tougher seed coats [9]. Similarly, adult *Leptoglossus occidentalis* (Hemiptera: Coreidae) will re-use a conspecific's feeding site when feeding through the thick shell of a pine nut [19]. However, feeding facilitation has not been well-studied in more generalist species, such as *L. zonatus*, that feed on host plants with a wide range of defences.

To test if facilitation by adults contributes to juvenile survival in *L. zonatus*, we raised bugs with either an adult or juvenile conspecific on a diet of pecan nuts, *Carya illinoinensis*, either with or without shells. The type of physical barrier that protects pecans changes as the nuts mature: they initially have a thick husk, which then breaks open as the shell inside hardens around the nut [20]. It is possible that insect herbivores might feed more easily on pecans earlier in the season, before the shells harden, but even then, there is still a substantial barrier protecting the nut in the form of the husk [21]. Despite these defences, pecans are readily consumed by multiple *Leptoglossus* species in parts of the United States [17], Mexico [22,23] and Brazil [24]. Furthermore, both juvenile and adult *L. zonatus* have been observed feeding on pecan nuts in the field [23]. And this species has been shown to feed on and damage pecans throughout various stages of nut maturation, even after the husk has opened and the shell has hardened ([22,23]; B. Ree 2023, personal communication). Nevertheless, we predicted that *L. zonatus* nymphs feeding on nuts with shells would have lower survival than those feeding on nuts without shells due to the difficulty of feeding through a hard shell. Therefore, mature pecans with hard shells provide a convenient food source to test feeding facilitation in *L. zonatus* as they are a natural food source for this species, but still present a considerable feeding challenge that can be manipulated experimentally (i.e. the shell can be removed).

We hypothesized that nymphs would re-use the feeding sites of larger conspecifics to facilitate feeding through barriers. As a result of this facilitation, we expected nymphs feeding on nuts with shells to have higher survival when housed with an adult than with another nymph in the same instar. Of course, nymphs may gain feeding-related or other benefits from conspecifics of any life stage. By comparing the survival of nymphs housed with an adult with those housed with a similarly sized nymph, we could isolate any added benefit provided by a larger conspecific with a longer rostrum. Alternatively, adults may also compete with nymphs for nutrients, in which case they would probably have a competitive advantage due to their longer rostrum and overall size. However, we expected competition to be minimal in our experiment as a single pecan nut provides sufficient nutrition for multiple bugs if access to the nut is not restricted by a shell.

## 2. Methods

**Figure 1.**
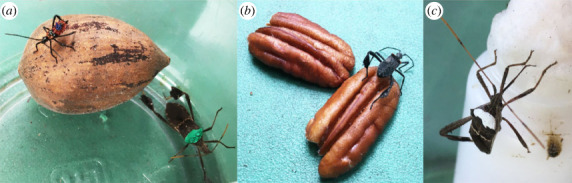
Photos of (left) a fourth instar standing on a pecan nut with its shell intact and an adult bug to the right of the nut, marked with green paint; (middle) a fifth instar walking over the two halves of a pecan nut without a shell; and (right) an adult bug marked with white paint, with its rostrum extended to drink from a water wick.

We collected wild *L. zonatus* from agricultural fields in northcentral Florida (USA) in the spring of 2019 and reared them in captivity in Gainesville, Florida for approximately five generations prior to starting this experiment. Bugs in this laboratory colony were fed a mixed diet of fruits and nuts and were allowed to mate freely. During the experiment, which ran from July to October 2020, bugs were housed in a covered outdoor location at ambient environmental conditions. We made treatment containers out of plastic deli cups (8 cm diameter base, 11 cm diameter top, 15 cm height) that each contained a single pecan nut (either with or without a shell), a water wick and two bugs (one focal and one non-focal; figure 1). Focal bugs were randomly assigned to one of four treatments: nut with shell + nymph (*N* = 30); nut with shell + adult (*N* = 30); nut without shell + nymph (*N* = 31); nut without shell + adult (*N* = 31).

All focal bugs, as well as the non-focal nymphs, were in their second instar (out of five) at the start of the experiment. We chose to start with second instars because this is the life stage at which they begin to feed [17]. To differentiate the focal and non-focal bugs in each container, we marked their pronotum using paint pens of different colours and re-marked nymphs after each moulting (approximately once per week). Cups were checked daily and the date at which each focal bug died or reached adulthood was recorded. Both pecans and non-focal bugs were replaced when needed due to pecan moulding or bug death. When replacing non-focal nymphs, we chose an individual that matched the current instar of the focal bug. We continued assigning bugs to experimental treatments from 1 July to 13 September and continued checking the cups daily until all bugs had died or eclosed as adults.

To test the effects of both the shell and conspecific life stage on nymph survival, we ran a Cox proportional hazards model using the *survival* package [25] and plotted survival curves using the *survminer* package [26] in R 4.3.0 [27]. The survival data were censored at 27 days after bugs emerged as second instars and were assigned to experimental treatments. Our model included the following independent factors: pecan shell (present or absent); conspecific life stage (nymph or adult); and the interaction between shell and conspecific life stage. Significance levels were evaluated using log likelihood ratio tests. We also calculated the proportion of bugs in each treatment that survived to adulthood.

## 3. Results

We observed *L. zonatus* of various life stages, including young nymphs (second and third instars) and adults, feed on pecans both with and without shells. Our observations included seeing the bugs extend their rostrum, fold back the outer labial sheath, and insert their stylets (inner mouthparts) through the pecan shell. To ensure their ability to feed on pecans with shells, we watched eight different nymphs feed continuously for at least 20 minutes without changing position or extracting their stylets from the nut.

**Figure 2.**
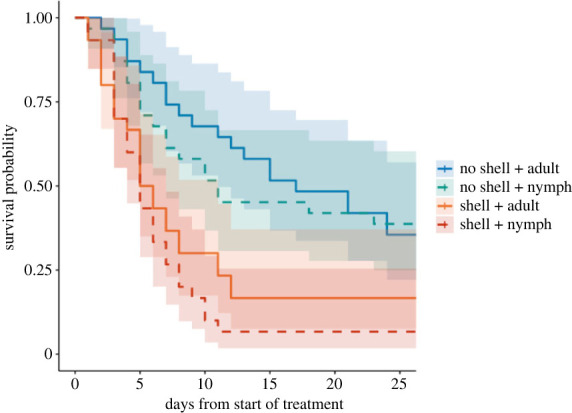
Survival curves showing the proportion of bugs that survived from the start of the second instar, when they were put into the experimental treatments, until 27 days later. The upper two curves are: no shell + adult (blue, solid); no shell + nymph (green, dashed). The lower two curves are: shell + adult (orange, solid); shell + nymph (red, dashed). A shaded 95% confidence interval surrounds each curve.

Despite this evidence of successful feeding, nymphs experienced lower survival when raised on pecans with shells than on pecans without shells ($\chi_{1}^{2}=18.66$, *p* < 0.001). Plotting the survival curves revealed that this survival difference between diet treatments was greatest during the first two weeks that bugs spent in the experiment, when they were mostly second and third instars (figure 2). By contrast, the presence of an adult versus juvenile conspecific did not affect the survival of the focal bug ($\chi_{1}^{2}=1.38$, *p* = 0.241). Furthermore, there was no interaction between the presence of a pecan shell and the life stage (adult or nymph) of the conspecific ($\chi_{1}^{2}=0.19$, *p* = 0.662).

Overall, 23 focal bugs survived from the start of their second instar stage (out of five) through adulthood. On average, these bugs took 28 days to complete their juvenile development and eclose as adults. The proportion of focal bugs that survived to adulthood in each treatment were: no shell + adult = 9/31 (29%); no shell + nymph = 8/31 (26%); shell + adult = 4/30 (13%); shell + nymph = 2/30 (7%).

## 4. Discussion

Previous research had found that *L. zonatus* nymphs feed on mature pecans in the field [22,23], and we confirmed that they are capable of feeding through a pecan shell even as young as second instars, which is the earliest stage at which nymphs begin to feed. However, our survival results demonstrated that structural feeding barriers like pecan shells can impose considerable limitations on the survival of juvenile *L. zonatus*. Second instars had lower survival on pecans with shells than on pecans without shells. However, this difference declined as the nymphs grew, so that for later instars, mortality rates were comparable across treatments (figure 2). These results parallel those of Cenzer [9], who found that juvenile soapberry bugs were most negatively impacted by feeding barriers during early developmental stages. It is possible that young nymphs expend more energy while feeding if they have to puncture a hard barrier, whereas this activity is probably less costly for a larger bug. Nymphs also may not be able to penetrate as deep inside a nut to access optimal nutrition if that nut is surrounded by a barrier. Furthermore, throughout juvenile development, nymphs are unable to fly, limiting their ability to find alternative host plants. Thus the defences of host plants are likely to be especially impactful in early life even in natural environments.

Contrary to our predictions, the presence of an adult conspecific compared with a juvenile conspecific did not significantly increase the survival probability of nymphs. In particular, our survival analysis revealed no interaction between conspecific life stage and shell presence, suggesting that adults did not impact developing nymphs differently depending on whether or not they faced a feeding barrier. There were minor differences in the proportion of bugs surviving from newly emerged second instars through adulthood based on whether they were raised with an adult (no shell = 29%, shell = 13%) or nymph (no shell = 26%, shell = 7%), but these differences were not significant in our survival analysis. Furthermore, we saw a much steeper mortality rate for second and third instars in treatments with pecan shells—both when housed with adults and with nymphs—compared with those without pecan shells (figure 2, around days 0–14). These early developmental stages are when feeding facilitation would be most likely to benefit juveniles [9], but there was no evidence of this happening in our experiment. Instead, our findings suggest that feeding facilitation by adults does not contribute substantially to *L. zonatus* nymphs' ability to feed through hard barriers such as nut shells.

Re-using feeding sites may be a more common strategy in more specialist herbivores that almost always feed through structural barriers, such as *L. occidentalis* [19] and *J. haematoloma* [9]. By contrast, *L. zonatus* has an extremely broad diet that, in addition to nuts, includes fruits with minimal structural defences, such as tomatoes, peppers and eggplants, as well as those with thick rinds, such as citrus, melons and pomegranates [17]. Because of variability in host plants across time and space, highly generalist feeders like *L. zonatus* may not be under consistent selection to be able to feed through physical barriers as juveniles. Examining this process in a standardized way across more herbivore species would help to clarify what relationship, if any, there is between diet breadth and juvenile reliance on feeding facilitation by adults.

As we only tested the feeding facilitation hypothesis using mature pecans, more studies are still needed to rule out potential facilitation in *L. zonatus* across other host plant species and food types. For example, it is possible that nearby feeding by adult *L. zonatus* could help nymphs feed through soft, thick barriers such as citrus rinds even if it does not help them feed through hard barriers like pecan shells. We also observed fairly low survival for young nymphs across all treatments in our experiment, which indicates that other aspects of our experimental set-up besides the pecan shell probably contributed to early mortality. In particular, we noticed fungal growth on some pecans, and although we replaced the affected nuts whenever this happened, fungal exposure may still have negatively impacted the health of the bugs. We also housed the bugs outside, so they were exposed to both high and low temperatures. While these environmental factors made our experiment more relevant to a natural context, they also probably contributed to the baseline mortality rate, making it more difficult to detect potential effects of feeding facilitation. Future research could pair field studies with controlled laboratory experiments to more comprehensively investigate this hypothesis.

Studying the strategies that enable juvenile herbivores to overcome physical feeding barriers is important to understanding the evolution of plant-herbivore interactions. Future studies should analyse how feeding ability and feeding behaviour change across each of the juvenile stages and into adulthood, taking the possibility of feeding facilitation into account, especially in herbivores that feed on structurally defended host plants. Documenting the species and conditions in which such facilitation is likely to occur is critical to predicting how herbivores might respond to future range shifts or changes in host plant communities.

## Data Availability

The dataset and code used in this study have been uploaded to the Dryad Digital Repository: https://doi.org/10.5061/dryad.wpzgmsbsm [28].
